# Positive Predictive Value of Contrast-Enhanced Fluid-Attenuated Inversion Recovery (FLAIR) Magnetic Resonance Imaging in Diagnosis of Meningitis Among Pediatrics Taking Cerebrospinal Fluid Analysis as Gold Standard

**DOI:** 10.7759/cureus.73356

**Published:** 2024-11-09

**Authors:** Sadia Shabbir, Maryam Sheikh

**Affiliations:** 1 Department of Radiology, The Children's Hospital Lahore, The University of Child Health Sciences, Lahore, PAK; 2 Department of Imaging and Radiology, The Children's Hospital Lahore, The University of Child Health Sciences, Lahore, PAK

**Keywords:** cerebral spinal fluid analysis, contrast enhanced fluid-attenuated inversion recovery, magnetic resonance imaging, meningitis, positive predictive value

## Abstract

Background and objective: Cerebrospinal fluid (CSF) analysis is the gold standard for meningitis diagnosis. It is invasive, time-consuming, and can inoculate infection. CSF analysis is not appropriate for many children without significant clinical suspicion, and delaying decision-making can have negative consequences. Therefore, medical research has long focused on speedy, non-invasive meningitis diagnosis. This study aimed to examine the positive predictive value (PPV) of contrast-enhanced fluid-attenuated inversion recovery magnetic resonance imaging (CE-FLAIR MRI) in meningitis diagnosis.

Materials and methods: It was a cross-sectional study that was conducted in the Department of Radiology, The Children’s Hospital in Lahore, from December 29, 2017, to June 28, 2018. The 198 patients were included of either gender with an age of 1 to 15 years and underwent CE-FLAIR MRI due to suspicion of meningitis. The gold standard for meningitis diagnosis was cerebrospinal fluid analysis, while CE-FLAIR MRI results were graded as true positives and false positives. The chi-square test was used. The *p*-value less than 0.05 was taken as statistically significant.

Results: The mean age of the patients was 4.18±2.48 years. The majority, 116 (58.6%), of the patients were aged below 5 years. There were 125 (63.1%) male and 73 (36.9%) female patients with a male-to-female ratio of 1.7:1. The diagnosis of meningitis using CE-FLAIR MRI was made in 187 (94.4%) patients with a PPV of 94.4%, which was further confirmed on CSF analysis. The PPV of CE-FLAIR-MRI across various subgroups based on the patient's age and gender were statistically insignificant, which showed consistency across all included age and gender groups (*p* > 0.05).

Conclusion: The contrast-enhanced FLAIR magnetic resonance imaging was found to have a high positive predictive value of 94.4% in the diagnosis of meningitis, taking cerebrospinal fluid analysis as the gold standard. CE-FLAIR MRI’s noninvasive and ionizing radiation-free characteristics advocate its preferred use in future clinical practice.

## Introduction

Meningitis is a major cause of febrile illness in childhood. Meningitis still affects children despite breakthroughs in diagnosis, treatment, and vaccines [1]. According to the World Health Organization (WHO), 1.2 million people globally contract bacterial meningitis, killing 135,000 [2]. Even with current treatment and intensive care, mortality is 5-10% [3]. Patients diagnosed with meningitis among the pediatric population who go untreated have a mortality rate of 100%. Age, early diagnosis, early treatment, duration, and microorganisms affect disease outcome. Preventing problems and improving outcomes requires early diagnosis and treatment [4].

The prevalence of meningitis in the South Asian population is 10.7%, and one study in Pakistan reported a prevalence of 11.11% [5,6]. Pakistan is among the top 10 countries with the most meningitis deaths, although worldwide burden estimates for low- and middle-income countries (LMICs), e.g., Pakistan, are limited. Despite having treatment approaches, mortality is still high in low-income countries (LICs) and LMICs, particularly among neonates and children. Diagnostics shortages in LICs and LMICs also cause broad uncertainty intervals in pathogen-specific incidence and death estimates [7].

The cardinal symptoms of meningitis are neck stiffness, headache, fever, altered mental status, and nausea. The typical signs and symptoms of pediatric meningitis on MRI consist of varying findings depending upon the type of meningitis and infective agents. Leptomeninges enhancement and intraventricular diffusion restrictions are signs of pyogenic bacterial meningitis. Moreover, sulcal signal changes and intraparenchymal alterations influencing the cortex and white matter have also been reported with diffusion-weighted imaging and FLAIR sequencing to detect changes [15]. The diagnostic performance of magnetic resonance imaging is varied among existing literature to diagnose meningitis among pediatric populations from the age of 1 to 12 years, depending upon the methodology and context of the technique used. MRI showed high specificity and moderate sensitivity to diagnosing meningitis infants. Kralik et al. (2022) found leptomeningeal enhancement to diagnose pyogenic meningitis using MRI. The authors reported MRI sensitivity ranges from 67.4% to 83.5% and specificity ranges from 92.3% to 95.7% [15]. In another study, Qureshi et al. (2020) carried out a study in Nishtar Hospital Multan, Pakistan, and found that CE-FLAIR MRI had shown a specificity of 83.67% and a sensitivity of 78.43% among children aged 2 to 12 years, showing diagnostic tool effectiveness as compared to lumbar puncture (CSF analysis) as the gold standard [16]. Moreover, Marchenko et al. (2021) provided insights in their study that multiparametric MRI consisting of diffusion-weighted imaging and MR spectroscopy has been proven to enhance the diagnostic performance of MRI, particularly among bacterial purulent meningitis pediatric patients [17]. Therefore, MRI is highly specific and increasingly sensitive, which provides a complementary role to the lumbar puncture (CSF analysis) technique for the diagnosis of pediatric meningitis patients.

In the existing literature, CSF (cerebrospinal fluid) analysis is the gold standard approach to diagnosing meningitis and increased raised intracranial pressure carries the risk of brain herniation [1,18]. However, CE-FLAIR is also found to be an effective and non-invasive diagnostic approach [19]. Although both diagnostic approaches have their own pros and cons, CSF analysis is invasive, and FLAIR is a non-invasive as well as safe approach [19].

Mushtaq et al. (2020) carried out a comparative observational study conducted in Islamabad, Pakistan, and found CE-FLAIR as an alternative tool to diagnose tuberculosis and meningitis and found a positive predictive value of 94.81% [20]. However, Ali et al. (2023) also carried out an observational study to diagnose tuberculosis meningitis patients. The authors found a 43% positive predictive value, which is contrastingly low compared to 94.81% [21]. There is a contrasting variation in the positive predictive value of contrast-enhanced FLAIR in predicting meningitis (94.81% and 43%). This may be due to the fact that one study used the single FLAIR sign as the diagnostic criteria and the second used FLAIR signs in >2 different areas. As the positive predictive value varies with the prevalence of the disease, more studies are needed to validate or challenge the findings further. Therefore, a positive predictive value of a non-invasive and safer modality could be determined because it can help in early diagnosing complicated cases, which can help in timely management by reducing the mortality and morbidity associated with meningitis.

The implication of this study is to find out the alternative, which should be non-invasive and safer. CSF analysis is the gold standard for meningitis diagnosis. However, it is invasive, time-consuming, and can lead to the inoculation of infection. Moreover, not every child can be subjected to CSF analysis without strong clinical suspicion, and the delay in decision-making can lead to an unwanted outcome. Therefore, quick and non-invasive diagnosis of meningitis has always been a focus of medical research. The recent studies also poised contrasting PPV for CE-FLAIR-MRI in the diagnosis of meningitis and also present the need to carry out further research to either validate or challenge the modality. Moreover, in central Punjab, the evidence was also limited, which necessitated the present study. The aim of this cross-sectional study is to determine CE-FLAIR MRI's PPV to diagnose meningitis using CSF analysis as the gold standard.

## Materials and methods

Study design and approval

It was a cross-sectional analytical study, which was approved by the Registration and Research Cell, College of Physicians and Surgeons Pakistan, with registration # PCPSP/REU/RAD-2016-075-2285.

Study setting and duration

The research was carried out in the Department of Radiology, The Children’s Hospital, Lahore, from December 29, 2017 to June 28, 2018.

Sample size

A sample size, taking a 95% confidence level and 2% margin of error, of the 198 cases was calculated using FLAIR MRI's expected positive predictive value of 97.95% in meningitis diagnosis, considering CSF as the gold standard [22].

Sampling technique

A consecutive non-probability sampling technique is used to select the patients.

Sample selection

Inclusion Criteria

Patients of either gender with 1-15 years of age and suspected of suffering from meningitis on FLAIR MRI were included. The diagnosis of patients was made on the following definition: patients having any two of the complaints, such as neck stiffness, fever > 102 °F, tonic colonic seizures, or visual disturbance, underwent MRI without contrast, and a second contrast-enhanced FLAIR image was taken and compared with the non-enhanced image. The presence of diffuse enhancement of any two (enhancement of sulci, cisterns, ventricles, or meninges) was labeled to have meningitis diagnosed by FLAIR MRI. However, meningitis on cerebrospinal fluid (CSF) analysis was labeled upon CSF analysis having total protein > 0.45 g/L, glucose > 0.5 mmol/L, and cell count > 15 per 3.2 nL or > 2 colonies on CSF examination after eosin hematoxylin staining. Patients whose blood relatives gave written informed consent to participate in the study were considered. 

Exclusion Criteria

Patients who were not diagnosed with meningitis on MRI and confirmed negative on CSF analysis were excluded. Patients who were sensitive to gadolinium-containing dye as per history and clinical examination were excluded. Patients who took at least one dose of antibiotic in the past week as per history and clinical record were also not included. Patients who developed papilledema on ophthalmoscopic examination and who underwent spinal surgery or lumbar puncture in the past month were also excluded. Patients with bleeding disorder 1 NR ≥1.2 as per laboratory findings at the time of lumbar puncture were not included in the study.

Data Collection

The 198 patients who met the aforementioned criteria and presented at the Radiology Department of The Children's Hospital, Lahore, were guided and explained the study after ethical review committee permission. Patients provided written informed consent and a comprehensive history. Data on patients' clinical profiles, such as signs, symptoms, blood glucose, CSF glucose, total protein, and cell count, were recorded in a self-structured questionnaire. The data or imaging interpretation was entered in a pro forma and MRI reporting file. The data were entered in IBM Corp. Released 2021. IBM SPSS Statistics for Windows, Version 28.0. Armonk, NY: IBM Corp. Moreover, Meningitis terminology and diagnostic methods are provided here.

MRI Procedure

An MRI non-contrast enhanced FLAIR scan was carried out of the brain from vertex to base of the skull at 1.5 tesla. The authors took TIW and FLAIR contrast-enhanced images. T1W contrast-enhanced imaging settings were: “TR: 500, TE: 7.8, FOV: 230 mm, image matrix: 224 x 256, slice thickness: 5 mm, slice interval: 1.5 mm, phase encoding direction: R to L, and acquisition time: 3 min 48 TR: 9000, TE: 109, TI: 2500, FOV: 230 mm, image matrix: 224 * 256, slice thickness: 5 mm, slice interval: 1.5 mm, phase encoding direction: R to L, acquisition time: 2 min, 08 sec, CE-FLAIR A weight-based computer-controlled injector administered gadolinium contrast medium at 0.2 mL/second to all individuals”. A radiologist consultant interpreted while monitoring images.

Cerebrospinal Fluid (CSF) Analysis

A lumbar puncture was performed by the consultant of the anesthesia department utilizing L4-L5 space under aseptic measures, and CSF was sent for analysis. Meningitis was labeled as per the operational definition. All the data was recorded along with the demographic details of the patient. All the MRI scans were performed on the same machine and were reported by the same consultant of the radiology department, all the lumber punctures were performed by the same consultant of the anesthesia department, and CSF analysis was done by the same consultant of the pathology department.

Statistical analysis

All data was entered and analyzed in IBM Corp. Released 2021. IBM SPSS Statistics for Windows, Version 28.0. Armonk, NY: IBM Corp. Numerical variables, e.g., age, are shown as mean ±SD. Gender and meningitis on CSF examination are presented by frequency and percentage. A percentage of positive predictive value was obtained using the following formula.

True positive

PPV=---------------------------------------------- x 100

True positive + false positive

True positive (TP) is defined as the patient having meningitis on FLAIR MRI later found to be true on CSF analysis. False-positive (FP) patients were having meningitis on FLAIR MRI, later found to be false on CSF analysis. The negative cases were not found, which is why they are not mentioned in the final analysis to make the analysis more comprehensive. The data is segmented by age and gender to address effect modifiers. A post-stratification chi-square test was used to rule out the impact of effect modifiers such as age and gender, with a p-value ≤0.05 considered significant.

Ethical statement

The research was approved by the Registration and Research Cell of the College of Physicians and Surgeons Pakistan (CPSP/REU/RAD-2016-075-2285). The cross-sectional study was carried out to determine diagnostic positive predictive value following the Declaration of Helsinki principles. The data was collected from the Radiology Department, The Children’s Hospital, Lahore. The confidentiality and anonymity of patients were also ensured. The informed consent was taken from the guardians or parents before enrolling them in the study. The participant's guardian or parents were described in the Urdu language as saying that the data would only be used for the beneficence of patients after de-identification. They had the right to leave the study at any time. Strengthening the Reporting of Observational Studies in Epidemiology (STROBE) guidelines were followed while writing up the manuscript.

## Results

The study included 198 patients aged 1-15 years (mean age: 4.18±2.48 years, ranging from 1.70 to 6.66 years). The majority of 116 (58.6%) patients were under 5. Table 1 shows 125 (63.1%) male and 73 (36.9%) female patients, with a male-to-female ratio of 1.7:1. 

**Table 1:** Baseline characteristics of the included patients (n=198)

Characteristics	Study Sample n=198
Age (years)	4.18±2.48
<5 years	116 (58.6%)
≥5 years	82 (41.4%)
Gender	
Male	125 (63.1%)
Female	73 (36.9%)

Meningitis was diagnosed by FLAIR MRI in 187 (94.4%) patients and confirmed by CSF analysis. The CE-FLAIR MRI in meningitis diagnosis with cerebrospinal fluid analysis as the gold standard had a PPV of 94.4% with 187 true positives and 11 false positives. The following formula is used to calculate PPV (Table 2). 

**Table 2:** Diagnosis of meningitis on cerebrospinal fluid analysis (n=198) TP: True positive, FP: False positive, PPV: Positive predictive value

Meningitis	Frequency (n)	Percent (%)
Yes (True Positive)	187	94.4
No (False Positive)	11	5.6
Total	198	100.0
Formula; TP = 187, FP = 11, PPV = 187 /187 + 11 = 94.4%		

The case outcome (true versus false positive) was post-stratified across demographics (age, gender) of patients. The positive predictive value of contrast-enhanced FLAIR-MRI across various subgroups based on the age and gender of patients was consistent and statistically insignificant (p>0.05). Therefore, the results or diagnostic positive predictive value is consistent among both age groups and for both genders (Tables 3, 4).

**Table 3:** Stratification of positive predictive value across various age groups (n = 198). *Chi-square test, observed difference was statistically insignificant, PPV: Positive predictive value

Age	n	Case outcome		Total	PPV	P-Value
		True Positive (n = 187)	False Positive (n = 11)			
<5	116	109	7	116	94.0%	0.726
		94.0%	6.0%	100.0%		
≥5	82	78	4	82	95.1%	
		95.1%	4.9%	100.0%		

**Table 4:** Stratification of positive predictive value across various genders (n = 198). Chi-square test, the observed difference was statistically insignificant, PPV: Positive predictive value

Gender	n	Case outcome		Total	PPV	P-Value
		True Positive (n = 187)	False Positive (n = 11)			
Male	125	118	7	125	94.4%	0.972
		94.4%	5.6%	100.0%		
Female	73	69	4	73	94.5%	
		94.5%	5.5%	100.0%		

## Discussion

This cross-sectional study comprised 198 patients with a mean age of 4.18±2.48 years (ranging, from 1.70-6.66 years). The majority of 116 (58.6%) patients were under 5 years of age. There is also a slight male predominance (63.1% male, 36.9% female) in including sample size with a male-to-female ratio of 1.7:1. The meningitis was diagnosed by FLAIR MRI in 94.4% of patients and confirmed by CSF analysis as the gold standard. FLAIR MRI had a positive predictive value of 94.4% (187 true positives and 11 false positives). After post-stratification across patient age and gender, the diagnostic performance of contrast-enhanced FLAIR-MRI was consistent among included different age and gender groups (p > 0.05).

The positive predictive value (PPV) of contrast-enhanced fluid-attenuated inversion recovery (FLAIR) magnetic resonance imaging is 94.4% in diagnosed meningitis patients. Similarly, Mushtaq et al. (2020) compared contrast-enhanced FLAIR as an alternative tool to diagnose tuberculosis meningitis and found a positive predictive value of 94.81% in diagnosed tuberculosis meningitis patients [20]. However, Khalid et al. (2023) reported 89.23% PPV of CE-FLAIR MRI in diagnosing infective meningitis [23]. Jawwad et al. (2022) and Wabasa et al. (2022) reported the PPV of contrast-enhanced FLAIR MRI as 87.6% and 88.8%, respectively [24,25]. All previously published literature also consistently reports somehow high PPV and sensitivity of the CE-FLAIR MRI that support its inclusion as a non-invasive and ionizing radiation-free diagnostic modality in routine MRI protocols for suspected cases of meningitis.

Moreover, the diagnostic performance of CE-FLAIR MRI was also superior in current literature in the diagnosis of different medical conditions. However, Khalid et al. (2023) also showed its sensitivity is 95.08%, specificity is 82.5%, and diagnostic accuracy is 90.1%, which makes it an authentic and reliable tool to diagnose infective meningitis patients in comparison to the CSF analysis as the gold standard test [23]. However, the CE-FLAIR MRI is also reported as a better modality in terms of specificity and sensitivity above 90% as compared to ultrasonography to diagnose myometrial anomalies in the brain [26]. Therefore, different diagnostic modalities’ performance must be compared in future research to ensure better safety, accuracy, and quick response to early diagnosis of the pathology for the benefit of patients’ health outcomes. It may also ensure evidence practice, further validating informed-based practice in clinical settings.

In this study, the meningitis patients, whose mean age was 4.18±2.48 years (1.70-6.66 years), were included. The CE-FLAIR MRI in this age pediatric population is also recommended and suggested due to the beneficial and better diagnostic capabilities reported in current literature. Capasso et al. (2023) also suggest CE-FLAIR MRI use due to its clearer visualization of meningitis as compared to traditional T1-W images because it helps to differentiate between enhancing meninges and cortical veins that appear hypointense on CE-FLAIR images [27]. Moreover, FLAIR MRI is also effective in assessing basal cistern enhancement and enhancement along the cerebellar folia, which plays a critical role in diagnosing meningitis [28]. Therefore, the integration of FLAIR MRI into routine diagnostic protocols to diagnose pediatric meningitis enhances the reliability and accuracy of detecting meningeal infections, which also improves outcomes of patients through early diagnosis efforts.

The mean age of patients suffering from meningitis in this study is 4.18±2.48 years. Similarly, Zghair et al. (2018) found somehow a mean age of 3.3±3.2 years has been reported in children suffering from meningitis in Iraq [29]. There is also a slight male predominance (63.1% male, 36.9% female) in including sample size with a male-to-female ratio of 1.7:1. In the current published literature, the male-to-female ratio in the prevalence of meningitis differed by type of meningitis, region, and age group. Bano K (2018) carried out a study in Pakistan and found a closer male-to-female ratio of 2:1 in males to females among patients of tuberculosis meningitis [30]. Alkassoum et al. (2024) in their national surveillance data for meningitis patients also reported a slight male predominance (1.30:1) [31]. However, Ayyaz Afzal et al. (2018) carried out an observational study in Multan, Karachi, and reported a slight female predominance. The authors reported that around 55% of tuberculosis and meningitis patients were females [32]. Green et al. (2020), in their meta-analysis to determine sex differences between meningitis patients, found that male-to-female predominance was found in younger age patients as compared to older age patients. Therefore, male-to-female predominance differed depending on the type, region, and age groups [30].

The diagnostic performance of contrast-enhanced FLAIR-MRI was consistent across different ages (1-15 years) and gender groups in children (p>0.05). The current existing literature is somehow limited to addressing this age-specific diagnostic performance consistency of CE-FLAIR MRI. However, Jawwad et al. (2022) also reported that there is no variance in diagnostic performance of CE-FLAIR MRI reported across 2-70 years of age among both genders. The FLAIR MRI diagnosis performance showed consistency among all age and gender groups, which is consistent with the findings of this study [25]. However, the study is observational in design, which cannot determine the cause. Therefore, future researchers are suggested to utilize causal-determining research design to either validate or challenge this consistency.

The CE-FLAIR MRI, due to its potential benefits, such as non-invasive and ionizing radiation, makes it a safer and more reliable tool. Although it is a valuable tool, CSF analysis as a gold standard limits reliance on it as an imaging tool alone. However, considering its benefits and potential as a valuable tool, it is a helpful tool among patients when there is negligible suspicion or there is no considerable diagnosis. Moreover, it is preferred, especially when CSF analysis is not feasible and contraindicated. FLAIR MRI is a valuable alternative tool that has greater implications for use in clinical practice with supporting evidence among pediatric populations of either gender with better safety and positive predictive value.

The following efforts were carried out to minimize bias and address potential limitations. It is a cross-sectional observational study design, but STROBE guidelines were used to enhance the robustness of the findings of this study. The inherent publication bias is tried to avoid by taking CSF analysis as a gold standard test to cross-check and monitor the false positive results. A main limitation of the present study was that it was a single-center experience due to the operator-dependent nature of radiological tools. Moreover, the study involves one experienced radiologist observer throughout the study. The authors suggest involving more than one observer in future studies to develop interobserver agreements on the evidence and findings. This may further strengthen the evidence interpretation by observing and countering the interpretation of FLAIR MRI images of meningitis patients. The inclusion of more than one radiologist may also be utilized in future research. Although the study is also based on experience from a single public center with significant patient turnover, However, the authors suggested a multi-center study to further validate the diagnostic performance of FLAIR MRI across diverse populations, which may enhance the generalizability of the findings.

## Conclusions

Contrast-enhanced fluid-attenuated inversion recovery (FLAIR) magnetic resonance imaging was found to have a high positive predictive value of 94.4% in the diagnosis of meningitis, taking cerebrospinal fluid analysis as the gold standard. Its noninvasive and ionizing radiation-free characteristics advocate its preferred use in clinical practice for quicker and more efficient diagnosis of meningitis. Future researchers must focus on conducting more regional studies based on multicentres rather than single centers to enhance the diversity of findings and further validate the evidence. Future studies are recommended with a more robust experimental design, and multicentre may increase the validity of evidence to ensure evidence-based practice and lessen the burden of disease by providing an efficient and noninvasive diagnostic approach.
